# Tongxinluo Prevents Endothelial Dysfunction Induced by Homocysteine Thiolactone *In Vivo* via Suppression of Oxidative Stress

**DOI:** 10.1155/2015/929012

**Published:** 2015-10-11

**Authors:** Yi Zhang, Tiecheng Pan, Xiaoxuan Zhong, Cai Cheng

**Affiliations:** ^1^Department of Surgery, The Third Affiliated Hospital of Jianghan University, Wuhan 430300, China; ^2^Division of Cardiothoracic Surgery, Tongji Hospital of Tongji Medical College, Huazhong University of Science and Technology, Wuhan 430030, China

## Abstract

*Aim*. To explore whether Chinese traditional medicine, tongxinluo (TXL), exerts beneficial effects on endothelial dysfunction induced by homocysteine thiolactone (HTL) and to investigate the potential mechanisms. *Methods and Results*. Incubation of cultured human umbilical vein endothelial cells with HTL (1 mM) for 24 hours significantly reduced cell viabilities assayed by MTT, and enhanced productions of reactive oxygen species. Pretreatment of cells with TXL (100, 200, and 400 *μ*g/mL) for 1 hour reversed these effects induced by HTL. Further, coincubation with GW9662 (0.01, 0.1 mM) abolished the protective effects of TXL on HTL-treated cells. In *ex vivo* experiments, exposure of isolated aortic rings from rats to HTL (1 mM) for 1 hour dramatically impaired acetylcholine-induced endothelium-dependent relaxation, reduced SOD activity, and increased malondialdehyde content in aortic tissues. Preincubation of aortic rings with TXL (100, 200, and 400 *μ*g/mL) normalized the disorders induced by HTL. Importantly, all effects induced by TXL were reversed by GW9662. *In vivo* analysis indicated that the administration of TXL (1.0 g/kg/d) remarkably suppressed oxidative stress and prevented endothelial dysfunction in rats fed with HTL (50 mg/kg/d) for 8 weeks. *Conclusions*. TXL improves endothelial functions in rats fed with HTL, which is related to PPAR*γ*-dependent suppression of oxidative stress.

## 1. Introduction

Hyperhomocysteinemia might play a role in the pathogenesis of vascular disorders and it is considered as an independent risk factor for atherosclerosis in 1969 [1]. Homocysteine occurs in human blood plasma in several forms, including the most reactive one which is HTL of a cyclic thioester, and represents up to 0.29% of total plasma homocysteine [2]. HTL reacts with proteins by acylation of free basic amino groups. In particular, the epsilon-amino group of lysine residues forms adducts and induces structural and functional changes in plasma proteins [3]. High levels of homocysteine impair endothelial function and cause endothelial damage in humans and in animal models [4, 5], indicating that the endothelial monolayer is very sensitive to changes in plasma homocysteine levels.

Tongxinluo (TXL) is a traditional Chinese compound prescription and has been approved by the State Food and Drug Administration of China in 1996 for the treatment of angina pectoris and ischemic stroke. Increasing evidence has indicated that the TXL has cardioprotective functions. Treatment with TXL is effective in lowering serum lipid levels, inhibiting plaque inflammation, and enhancing stability of vulnerable plaques [6]. It can also reduce myocardial no-reflow and ischemia-reperfusion injury and modulate vascular endothelial function [7]. Based on the aforementioned studies, we tested the hypothesis that TXL may produce protective effects on endothelial dysfunction induced by high homocysteine via suppression of oxidative stress. Here, we reported that pharmacological activation of peroxisome proliferator-activated receptor gamma (PPAR*γ*) by TXL improves endothelial function in rats with hyperhomocysteinemia.

## 2. Materials and Methods

### 2.1. Materials

TXL was from Shijiazhuang Yiling Pharmaceutical Company, China. GW9662 (2-Chloro-5-nitro-N-phenylbenzamide, Cat.: M6191, purity >98%), pyrrolidine dithiocarbamate (PDTC), dihydroethidium (DHE), apocynin, acetylcholine (Ach), sodium nitroprusside (SNP), and phenylephrine (PE) were purchased from Sigma Company. Commercial kits for determinations of malondialdehyde (MDA) and superoxide dismutase (SOD) activity were from Cayman Company.

### 2.2. Components, Preparation, and Chemical Analysis of TXL

The herbal drugs of TXL were authenticated and standardized on marker compounds according to the Chinese Pharmacopoeia 2005 as described previously [8]. TXL contains 12 medicinal components:* Panax ginseng* C.A. Mey, 1.677;* Ziziphus jujube Mill. *var.* spinosa*, 1.173%;* Paeonia lactiflora Pall.*, 1.558%;* Santalum album L.*, 0.354%;* Dalbergia odorifera T. Chen*, 4.005%;* Boswellia carteri Birdw.*, 5.927%;* Borneolum syntheticum*, 3.626%;* Scolopendra subspinipes mutilans L. Koch*, 3.623%;* Buthus martensii Karsch*, 18.111%;* Steleophage plancyi*, 18.111%;* Hirudo nipponica*, 27.330%;* Cryptotympana pustulata Fabricius*, 18.111%. They were ground to superfine powder with the diameter of 10 *μ*m or less by a Micronizer and prepared as capsules. To reduce the dose variability of TXL capsule among different batches, the species, origin, harvest time, medicinal parts, and concocted methods for each component were strictly standardized. Moreover, high performance liquid chromatography was applied to quantitate the components of the TXL capsule. Five major components of the aqueous extract of tongxinluo capsule included* paeoniflorin* (6.142 mg/g),* ginsenoside Rg1* (0.884 mg/g),* ginsenoside Rb1* (0.730 mg/g),* jujuboside A* (0.661 mg/g), and* jujuboside B* (0.646 mg/g). Besides, another two peaks on the gas chromatogram fingerprint of TXL capsule were identified, which are* isoborneol *and* borneol*.

### 2.3. Animals

Male Sprague-Dawley rats (8 ± 2 weeks old, 180 ± 20 g) were purchased from the Center of Experiment Animals, Central South University (Changsha, China). Rats were housed in temperature-controlled cages with a 12-hour light-dark cycle. The animal protocol was reviewed and approved by Jianghan University and Huazhong University of Science and Technology.

### 2.4. Cell Culture

Human umbilical vascular endothelial cells (HUVECs) from America Type Collection Center (ATCC) were grown in endothelial cells basal medium (Clonetics Inc., Walkersville, MD) supplemented with 2% FBS, penicillin (100 U/mL), and streptomycin (100 *μ*g/mL). In all experiments, cells were between passages 3 and 8. All cells were incubated at 37°C in a humidified atmosphere of 5% CO_2_ and 95% air. Cells were grown to 70–80% confluency before being treated with different agents.

### 2.5. Organ Chamber

Organ chamber study was performed as described previously [9]. Rats were sacrificed under anesthesia by intravenous injection with pentobarbital sodium (30 mg/kg). The descending aorta isolated by removing the adhering perivascular tissue carefully was cut into rings (3-4 mm in length). Aortic rings were suspended and mounted to organ chamber by using two stainless clips. The rings were placed in organ baths filled with Krebs buffer of the following compositions (in mM): NaCl, 118.3; KCl, 4.7; MgSO_4_, 0.6; KH_2_PO_4_, 1.2; CaCl_2_, 2.5; NaHCO_3_, 25.0; EDTA, 0.026; pH 7.4 at 37°C and gassed with 95% O_2_ plus 5% CO_2_. Before contraction, a tension of 2 g was given to the aorta ring for 90 minutes. During this period, Krebs solution was changed every 15 min. After the equilibration, aortic rings were challenged with 60 mM KCl. After washing and another 30 minutes equilibration period, contractile response was elicited by PE (1 *μ*M). At the plateau of contraction, accumulative Ach (0.01, 0.03, 0.1, 0.3, 1, and 3 *μ*M) or SNP (0.01, 0.03, 0.1, 0.3, 1, 3, and 10 *μ*M) was added into the organ bath to induce endothelium-dependent or -independent relaxation.

For* ex vivo* experiments, the rings were contracted by PE (1 *μ*M) and then dilated with cumulative concentrations of Ach (0.01–3 *μ*M) to assess the integrity of the endothelium. The ring whose maximal relaxation induced by Ach (3 *μ*M) is over 80% was considered to have intact endothelium and was used in the following study. Then, the rings were pretreated with TXL (100, 200, 400 *μ*g/mL) for 30 minutes followed by addition of HTL (1 mM) for 90 minutes. After washing, Ach-induced endothelium-dependent relaxation and SNP-induced endothelium-independent relaxation were assayed, respectively. At the end of the experiments, the aortic rings were collected in liquid nitrogen for measurements of NO and MDA after homogenization.

### 2.6. Measurements of MDA Content, SOD Activity, and Nitric Oxide (NO) Level

The contents of MDA content, SOD activity, and NO level in aortic tissues or blood were assayed by using commercial kits following the recommended protocol.

### 2.7. Evaluation of Cell Viability

Cell viability was assayed by using 3-(4,5)-dimethylthiahiazo(-z-y1)-3,5-di-phenytetrazoliumromide (MTT) as described previously [10]. Cells were seeded into 96-well plate at the density of 10000/mL and incubated for 24 hours. After treatment, 10 *μ*L MTT (5 mg/mL) was added into cultured medium in each well for 2–4 hours until purple precipitate is visible. After the removal of culture medium, 75 *μ*L dimethyl sulphoxide was added to each well, leaving the cells at room temperature in the dark for 2 hours. The absorbance at 570 nm was recorded.

### 2.8. Detection of ROS

ROS productions in cultured cells were assayed by measuring the DHE fluorescence as described previously [11]. Briefly, before the end of the treatment, 10 *μ*M DHE was added to the medium, incubated for 30 min at 37°C, and then washed with PBS twice. The image was taken by fluorescent microscope. The DHE fluorescent intensity was recorded by fluorescent reader at the wave of excitation (485 nm) and emission (545 nm). Control was set up as 100%.

### 2.9. Statistical Analysis

Data are reported as mean ± SEM. All data were analyzed with the use of 1- or 2-way ANOVA followed by multiple *t*-tests, and *P* < 0.05 were considered statistically significant.

## 3. Results

### 3.1. TXL Dose-Dependently Normalizes Cell Viabilities in HTL-Treated Endothelial Cells

**Figure 1:**
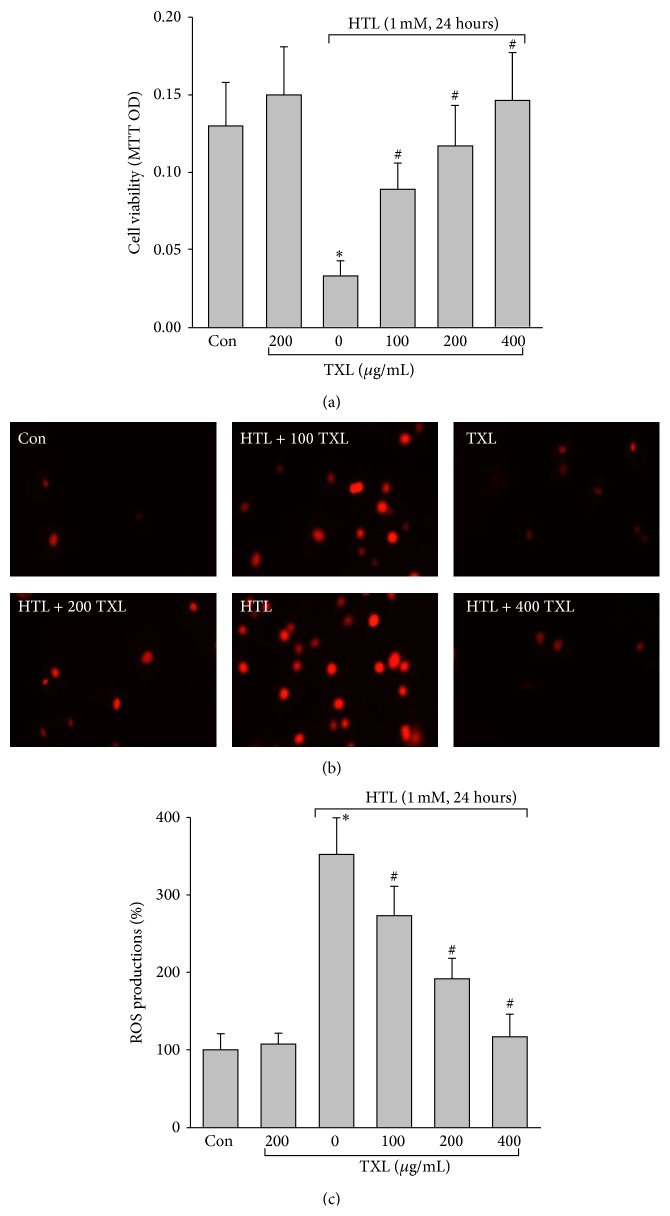
TXL dose-dependently suppresses HTL-induced oxidative stress and improves cell viabilities in cultured HUVECs. Cultured HUVECs were pretreated with TXL (100, 200, and 400 *μ*g/mL) for 1 hour and then incubated with HTL (1 mM) for 24 hours. (a) Cell viability was measured by MTT. (b) Intracellular ROS productions were detected by DHE fluorescence. (c) Quantitative analysis of ROS productions. All data were expressed as mean ± SEM. *N* is 3 in each group. ^*∗*^ *P* < 0.05 versus Con, ^#^ *P* < 0.05 versus HTL alone.

We firstly investigated whether HTL, which is the most reactive one of homocysteine, affected cell viabilities in cultured HUVECs. As shown in Figure 1(a), incubation of HUVECs with HTL (1 mM) for 24 hours dramatically reduced cell viabilities detected by MTT assay, indicating that detrimental effects of homocysteine are related to the high reactivity of HTL. We next examined whether TXL protected endothelial cells against HTL. As depicted in Figure 1(a), TXL alone did not affect endothelial cell viabilities but dose-dependently reversed cell viabilities reduced by HTL.

### 3.2. TXL Dose-Dependently Suppresses HTL-Induced Oxidative Stress in Cultured Endothelial Cells

We then investigated that TXL via suppression of oxidative stress maintains the normal phenotypes of vascular endothelial cells. As expected in Figures 1(b) and 1(c), incubation of HUVECs with HTL (1 mM) for 24 hours remarkably increased ROS productions in cultured cells. However, preincubation of these cells with TXL inhibited the enhancements of ROS productions induced by HTL in a dose-dependent manner. Taking these together, it indicates that TXL protects cell viabilities reduced by HTL, which is possibly related to suppression of oxidative stress.

### 3.3. TXL via PPAR*γ* Protects HTL-Treated Endothelial Cells

**Figure 2:**
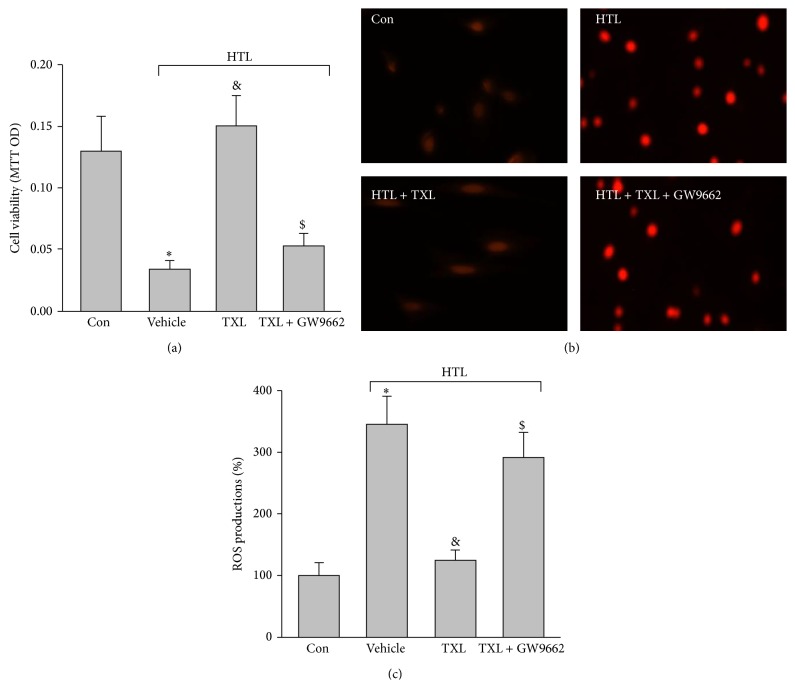
Inhibition of PPAR*γ* abolished TXL-suppressed oxidative stress and maintains cell viability in HTL-treated cells. Cultured HUVECs were pretreated with TXL (200 *μ*g/mL) for 1 hour and then incubated with HTL (1 mM) for 24 hours in presence of GW9662 (0.01 mM). Cell was subjected to assay: (a) cell viability by MTT and (b) intracellular ROS productions by DHE fluorescence. (c) Quantitative analysis of ROS productions. All data were expressed as mean ± SEM. *N* is 3 in each group. ^*∗*^ *P* < 0.05 versus Con, ^&^ *P* < 0.05 versus HTL alone, and ^$^ *P* < 0.05 versus HTL + TXL.

Activation of PPAR*γ* by agonists has been reported to produce protective effects on oxidative stress in endothelial cells [12, 13]. We next determined whether TXL via activation of PPAR*γ* provided beneficial effects in endothelial cells by using GW9662 which is a specific antagonist of PPAR*γ* to disrupt PPAR*γ* signaling [14]. As expected, inhibition of PPAR*γ* by GW9662 significantly abolished the protective effects of TXL on the improvement of cell viabilities (Figure 2(a)) and reductions of ROS productions (Figures 2(b) and 2(c)) in dose-dependent manner. Taking these data together, it indicates that TXL via PPAR*γ* activation suppresses oxidative stress and protects cell viabilities.

### 3.4. TXL via PPAR*γ* Preserves Endothelium-Dependent Relaxation Impaired by HTL in Mice Isolated Aortic Rings

**Figure 3:**
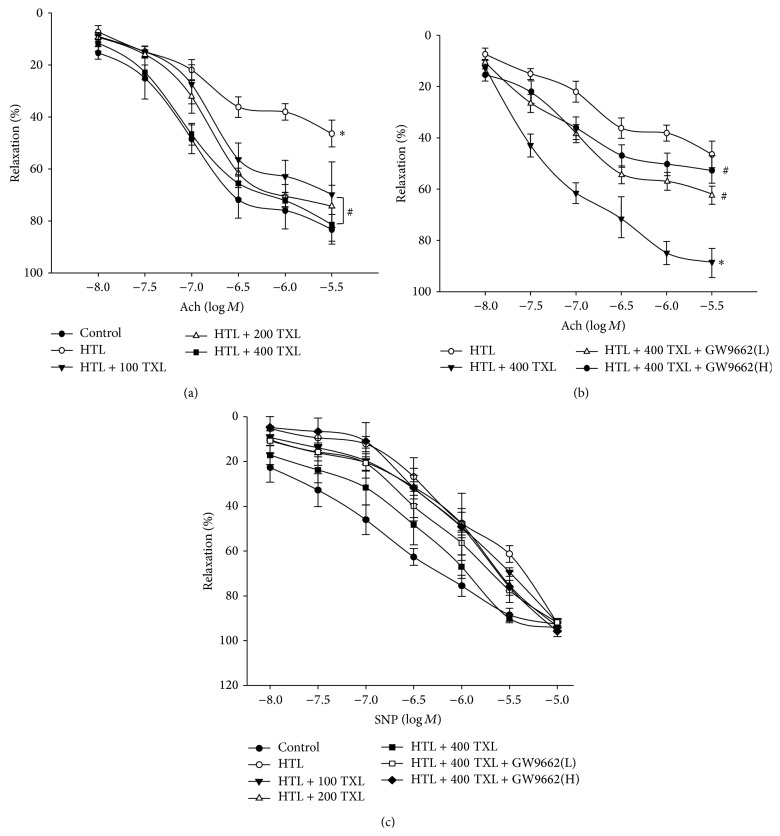
TXL via PPAR*γ* prevents from the impairment of endothelium-dependent relaxation induced by HTL in isolated rat aortas. (a) The isolated rat aortic rings were exposed to HTL (1 mM) for 1 hour after preincubation with TXL (100, 200, and 400 *μ*g/mL) for 30 minutes. The endothelium-dependent relaxation induced by acetylcholine (Ach) was assayed by organ chamber. All data were expressed as mean ± SEM. *N* is 5 in each group. ^*∗*^ *P* < 0.05 versus Control and ^#^ *P* < 0.05 versus HTL alone. (b) The isolated rat aortic rings were exposed to HTL (1 mM) for 1 hour after preincubation with TXL (400 *μ*g/mL) for 30 minutes with GW9662 (0.01, 0.1 mM). The endothelium-dependent relaxation induced by acetylcholine (Ach) was assayed by organ chamber. All data were expressed as mean ± SEM. *N* is 5 in each group. ^*∗*^ *P* < 0.05 versus HTL alone, ^#^ *P* < 0.05 versus HTL + TXL. (c) Endothelium-independent relaxation was assayed in all groups by using sodium nitroprusside (SNP).

We then performed* ex vivo* experiments to test whether TXL protected vascular endothelial functions by incubating isolated mice aortic rings with HTL. Exposure of aortic ring to HTL dramatically impaired Ach-induced endothelium-dependent relaxation (Figure 3(a)). Similar to* in vitro* observations from cultured cells, TXL dose-dependently reversed Ach-induced endothelium-dependent relaxation in aortic rings incubated with HTL (Figure 3(a)), suggesting TXL functions as a protector on vascular endothelium.

We next determined whether TXL via activation of PPAR*γ* provided beneficial effects in endothelial cells by using GW9662 [14]. As expected, inhibition of PPAR*γ* by GW9662 significantly abolished the protective effects of TXL on Ach-induced endothelium-dependent relaxation (Figure 3(b)), indicating that TXL via PPAR*γ* activation protects endothelial function. In addition, SNP-induced endothelium-independent relaxation was not altered in all groups (Figure 3(c)), suggesting that the protective effects produced by TXL on vascular function are limited to endothelium but not to vascular smooth muscle.

### 3.5. TXL via PPAR*γ* Reserves Redox State in Aortas, Which Is Disturbed by HTL

**Figure 4:**
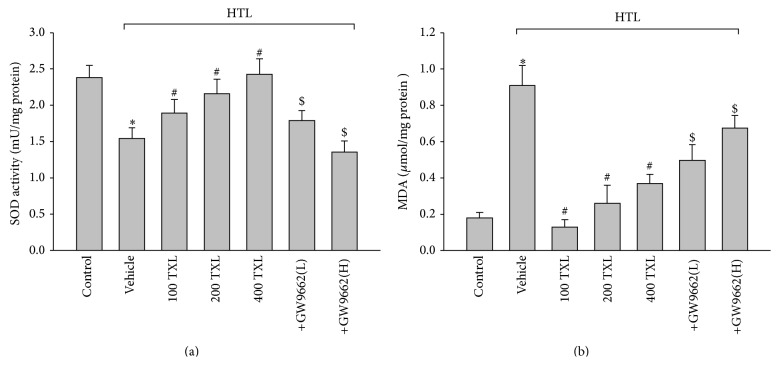
TXL via activation of PPAR*γ* suppresses oxidative stress in isolated rat aortas. The isolated rat aortic rings were exposed to HTL (1 mM) for 1 hour after preincubation with TXL (100, 200, and 400 *μ*g/mL) for 30 minutes. Aortas treated with 400 *μ*g/mL TXL were in the presence of GW9662 (0.01, 0.1 mM). Homogenates of aortic tissues were subjected to assay (a) SOD activity and (b) MDA content by commercial kits. All data were expressed as mean ± SEM. *N* is 5 in each group. ^*∗*^ *P* < 0.05 versus Control and ^#^ *P* < 0.05 versus HTL alone, and ^$^ *P* < 0.05 versus HTL + 400 TXL.

Decreased NO bioavailability, which is due to the decreased NO production or aberrant conversion of NO to ONOO^−^ by ROS, contributes to impairment of Ach-induced endothelium-dependent relaxation in cardiovascular system [15]. We then examined whether HTL maintains normal redox state in rat isolated aortic rings. We found that HTL dramatically decreased SOD activity (Figure 4(a)) and increased the content of MDA (Figure 4(b)), which is formed when ROS reacts with polyunsaturated fatty acid chain in membrane lipids [16]. However, pretreatment of cells with GW9662 significantly abolished TXL-rescued abnormalities in HTL-incubated aortas, suggesting that TXL via PPAR*γ*/SOD-MDA reserves the normal balance of antioxidative system.

### 3.6. Administration of TXL Improves Endothelial Function in Rats Fed with HTL

**Figure 5:**
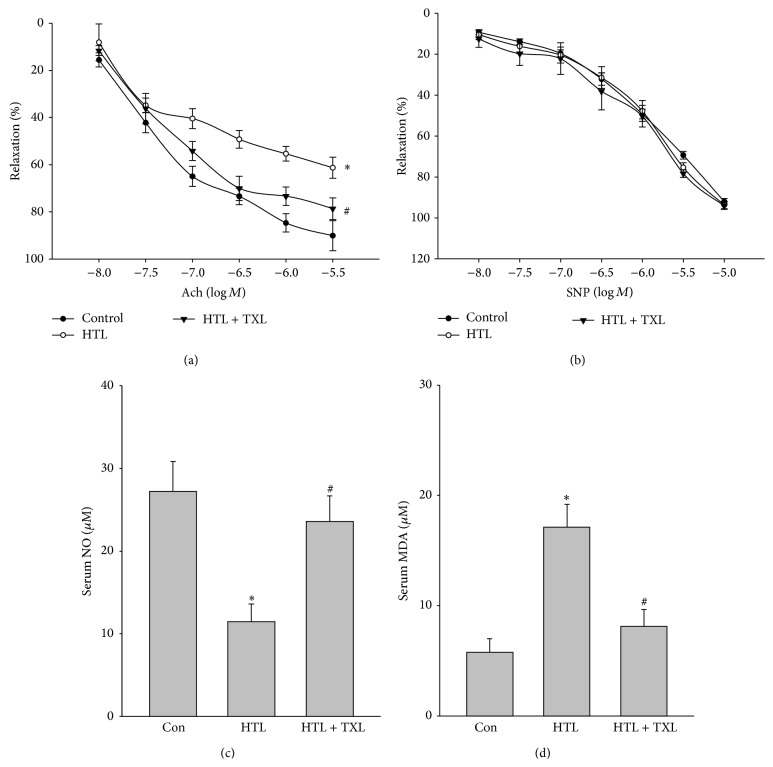
TXL suppresses oxidative stress and improves endothelial functions in rats fed with HTL* in vivo*. The rats were intragastric gavage HTL (50 mg/kg/d) and received administration of TXL (1.0 g/kg/d) for 8 weeks. At the end of experiments, rats were sacrificed under anesthesia. Artery from descending aorta was subjected to assay (a) endothelium-dependent relaxation induced by acetylcholine (Ach) and (b) endothelium-independent relaxation induced by sodium nitroprusside (SNP) in organ chamber. Blood was collected to assay serum level of (c) NO by Griess method and (d) MDA by TBA method. All data were expressed as mean ± SEM, 5–10 rats in each group. ^*∗*^ *P* < 0.05 versus Con and ^#^ *P* < 0.05 versus HTL alone.

We then performed* in vivo* experiments to confirm whether TXL improves vascular endothelial functions in rats with hyperhomocysteinemia. Homocysteine circulates as different species, mostly protein bound, with approximately 1% as its reduced form and the cyclic thioester HTL. Despite the fact that the level of plasma thiolactone is being markedly low, detrimental effects of homocysteine are related to the high reactivity of HTL [17]. We fed rats with HTL (50 mg/kg/day) for 13 weeks to mimic the model of hyperhomocysteinemia-induced endothelial dysfunction. Endothelial function was determined by measuring the vascular dilation induced by Ach. As shown in Figure 5(a), HTL significantly inhibited Ach-induced vascular relaxation. Importantly, the reduction of Ach-induced vascular relaxation was reversed by the administration of TXL. Both HTL and TXL did not change SNP-induced vessel relaxation (Figure 5(b)), suggesting that the improvement of TXL on vascular bioactivity in HTL-fed rats is due to maintaining endothelial function.

### 3.7. Administration of TXL Suppresses Oxidative Stress in Rats Fed with HTL

We finally determined whether TXL preserves the normal redox state in HTL-fed rats. As expected, HTL induced the alternations, such as decreased serum NO level (Figure 5(c)), and increased serum levels of MAD (Figure 5(d)). All these defects induced by HTL were normalized by the administration of TXL. This evidence indicates that the* in vivo* protective effects of TXL may be related to suppression of oxidative stress.

## 4. Discussions

The present study firstly demonstrates that HTL* in vitro* or* in vivo* causes accelerated oxidative stress and endothelial dysfunction, all of which are abrogated by TXL. Mechanistically, the protective effect of TXL on vascular function is attributable to PPAR*γ* activation, resulting in suppressions of oxidative stress. In this way, TXL normalizes the redox state in endothelial cells and protects endothelial function in hyperhomocysteinemic rats.

The major finding in this paper is that TXL prevents HTL-induced endothelial dysfunctions. Recent studies have found that the conversion of homocysteine into HTL plays a critical role in the progress of cardiovascular diseases in patients with hyperhomocysteinemia. In this present study, we used HTL to treat isolated aortic ring* ex vivo* or rats* in vivo*, by which both impaired Ach-induced endothelium-dependent relaxation, which is consistent with our previous study [18]. This supports the observation that detrimental effects of homocysteine are related to the high reactivity of HTL, though the level of plasma thiolactone is very low. Most importantly, HTL-induced endothelial dysfunction both* ex vivo* and* in vivo* was reversed by TXL, which is in dose-dependent manner. Collectively, our results suggest that TXL functions as a protector of endothelial cells. This discovery is also supported by several published studies done in cultured endothelial cells [19, 20] or animals [21, 22], which have shown that TXL protects endothelial function. However, a recent study on human reported that TXL caused endothelial dysfunction in normal volunteers. Of course, the reason for this discrepancy between healthy and hyperhomocysteinemia needs further investigations.

In summary, these studies support a novel function of TXL which activates PPAR*γ* to suppress NF-*κ*B -dependent nicotinamide-adenine dinucleotide phosphate hydrogenase NAD(P)H oxidase. This, in turn, inhibits oxidative stress in endothelial cells, leading to improvement of endothelia function. The finding that TXL attenuates endothelial dysfunction induced by HTL through suppression of oxidative stress may have broad applications for cardiovascular diseases, since endothelial dysfunction is a common character at the beginning and in the progress in a number of vascular diseases including atherosclerosis [23, 24] and diabetes [25]. Thus, TXL may be a useful drug for more effective treatment of atherosclerosis and hypertension.
